# Predicting aggressive outcome in T1N0M0 breast cancer

**DOI:** 10.1038/sj.bjc.6601948

**Published:** 2004-06-15

**Authors:** P Kronqvist, T Kuopio, M Nykänen, H Helenius, J Anttinen, P Klemi, and South-Western Finland Breast Cancer Group

**Affiliations:** 1 Departments of Pathology and Statistics, University of Turku, Turku, Finland; 2 Jyväskylä Central Hospital, Jyväskylä, Finland

**Keywords:** breast cancer, tumour size, prognosis

## Abstract

Despite the excellent overall prognosis, unpredictable breast cancer recurrences and deaths also occur among T1N0M0 patients. We have evaluated clinically applicable methods for identifying aggressive outcome in T1N0M0 breast cancer. The material is based on aggressive T1N0M0 invasive ductal and lobular carcinomas diagnosed in Turku University Hospital and Jyväskylä Central Hospital, Finland, during 1987–1997. We studied all the T1N0M0 breast cancers that had led to recurrency or death (*n*=21, 95% T1cN0M0) during the follow-up period (4–14 years). The study is based on statistical analyses of matched case–control data in which the prognostic factors of each individual patient with aggressive disease were compared with control patients (*n*=45) individually matched by tumour size, age at diagnosis, histological type of tumour and length of follow-up. The cancer cases were examined for clinically applicable conventional and immunohistochemical pathologic prognostic factors. High Ki-67 immunopositivity was the strongest prognosticator of breast cancer death or recurrence in T1N0M0 breast cancer. Also, high p53 immunopositivity, low oestrogen receptor immunopositivity and Her-2/neu oncogene amplification by chromogen *in situ* hybridisation were reliable indicators of unfavourable outcome. Our statistical methods also allowed us to determine for the present material a range of clinical significance for each immunohistochemical prognostic feature with the associated relative risk for breast cancer death and recurrence. The paper suggests guidelines for predicting aggressive outcome in T1N0M0 breast cancer.

In recent years, mammographic screening has made it possible to detect increasingly small invasive breast carcinomas and reduce the mortality of the disease (Anderson *et al*, 2000; Heimann *et al*, 2002; Louwman *et al*, 2002; Klemi *et al*, 2003; Tabar *et al*, 2003). Small tumour size and axillary lymph node negativity (T1N0M0) are significant indicators of excellent outcome in invasive breast cancer justifying local treatment as the only form of therapy (Bergh and Holmquist 2001; Colpaert *et al*, 2001; Isaacs *et al*, 2001; Morabiot *et al*, 2003). However, unpredictable breast cancer recurrences and deaths occur also among the group of patients with T1N0M0 breast cancers. Identifying aggressive disease and unfavourable outcome is essential for those T1N0M0 breast cancer patients who would benefit from adjuvant therapy.

In the present study, we analyse the strength of association between clinical prognostic variables and aggressive cancer in order to determine clinically applicable guidelines for identifying aggressive outcome in T1N0M0 invasive breast cancer. Usually in this kind of study approach, one must be prepared for huge numbers of specimens and exhaustive work in order to adjust the effect of the relevant confounders. We have solved this problem by strict data collection. The power of this study is in the matched case–control design of the data and the sophisticated statistical methods that allow us to evaluate efficiently the main prognostic factors and at the same time to take into account the prior well-known central confounding variables. The approach of individually matched case–control design shows that by elaborating conventional prognostic factors – Ki-67 proliferation index, p53 proto-oncogene expression, oestrogen hormone receptor and Her-2/neu proto-oncogene amplification detected by *in situ* hybridisation – with quantitative and statistical methods it is possible to intensify the prognostication of T1N0M0 breast cancer.

## MATERIALS AND METHODS

### Patient material

The material was analysed comparing two patient groups. The first group (aggressive cancers, *n*=21) involved all the aggressive T1N0M0 patients dead of breast cancer or detected with recurrency in two Finnish central hospitals during 1987–1997 (follow-up 4–14 years). These patients were individually matched with T1N0M0 patients alive of breast cancer with no detectable recurrency during the follow-up period (control cancers, *n*=42). The control cancers were selected as accurately as possible in order to match each of the individual aggressive cancer patient by tumour size, age at diagnosis and histological type of tumour. According to our selection rules, an aggressive cancer patient and the respective control cancer patients were allowed to differ no more than 2 mm in tumour diameter and no more than 8 years in age at diagnosis. Moreover, in order to avoid the influence of the length of follow-up time on the results, the survival time of each individual control cancer patient was determined equal to or longer than that of the corresponding individual aggressive cancer patient. Table 1

**Table 1:** Clinicopathological characteristics of the patient material (*n*=66)

**Variable**	**Aggressive cancers**	**Matched control cancers**
Tumor size (mm)	14.2	14.8
Mean	4–20	7–20
Range		
		
Age (year)	56.9	53.6
Mean	40–73	39–71
Range		
		
Follow-up (years)	5	8
Mean	4.7–11.7	4.7–14.7
Range		
		
Histological type	96.3	84.4
Ductal (%)	3.7	15.6
Lobular (%)		
		
Histological grade	12.2	32.6
I (%)	61.5	53.5
II (%)	19.3	13.9
III (%)		

shows the characteristics of groups of aggressive cancer and control cancer cases.

All patients had been treated with radical or modified radical mastectomy or conservation surgery with axillary evacuation. None of the patients had received preoperative radiation therapy or preoperative adjuvant treatment. The histological grading of carcinomas was performed according to Elston *et al* (1998). The number of axillary lymph nodes examined was recorded when obtainable from patient records. Complete follow-up histories and peri-operative tissue specimens from the primary tumours were available for each patient. The follow-up examination was carried out every 3 months during the first postoperative year, every 6 months during the second and third postoperative years and thereafter yearly, until 5 years of follow-up was completed. The mean follow-up period was 7 years (range from 4 years and 9 months to 14 years and 9 months).

Clinical follow-up investigation was completed by chest and bone radiographs, soft tissue ultrasound examinations and laboratory tests reflecting bone and liver metabolism. Recurrent disease was verified by cytological and histological samples or radiographs in case of bone metastasis. The causes of death were based on autopsy reports, death certificates and patient files from the Finnish Cancer Registry.

### Immunohistochemistry

The primary diagnosis of invasive ductal or lobular breast carcinoma was verified in van Gieson-stained slides and the corresponding areas of 5 *μ*m thick sections were stained for oestrogen (ER) and progesterone (PR) receptors, Ki-67 proliferation index and p53 oncogene. Immunohistochemical stainings (IHC) were performed with the TechMate 500 immunostainer and a peroxidase/diaminobenzidine (DAB) multilink detection kit (DAKO, Denmark), which is based on an indirect streptavidin–biotin method. Antigen retrieval was carried out using a microwave oven. Monoclonal antibodies against human ER *α*, Ki-67 antigen and p53 oncogene were supplied by Dako (DAKO, Denmark) and applied with dilutions 1 : 40, 1 : 100 and 1 : 300, respectively. Monoclonal antibody against human PR (dilution 1 : 20) was provided by Novocastra (Novocastra Laboratories Ltd, UK). Slides were counterstained with Mayer's haematoxylin.

### Chromogenic *in situ* hybridisation

Her-2/neu proto-oncogene was detected by chromogenic *in situ* hybridisation (CISH). CISH was performed on 3 *μ*m formalin-fixed paraffin-embedded tissue sections verified with cancer tissue as described above. Pretreatment of the sections was performed in citric acid and microwave oven. After the pretreatment, the specimens were digested with acidic pepsin solution. Digoxigenin-labelled c-erbB-2 probe (SPOT-Light HER2 DNA Probe, Zymed Inc., USA) was hybridised on the sections overnight and the probes were detected with horseradish peroxidase/diaminobenzidine system by the SPOT-Light CISH Polymer Detection Kit (Zymed Inc., USA). Finally, the sections were counterstained with Mayer's haematoxylin.

### Interpretation of IHC and CISH

Interpretations of IHC were standardised in evaluation sessions between two pathologists (PK and PK). Special consideration was placed on the selection of fields for evaluating IHC in order to ensure validity and reproducibility of results. First, in each case, we chose the representative slide, placing special emphasis on the preservation of histological details. Next, we identified the area of the actively proliferating cells at the border of the most cellular part of the tumour, rejecting areas showing necrosis and inflammation. Interpretation was performed by assessing with × 40 objective three separate evaluation areas with a total of 300 malignant cells. The fractions of positive cells (in percentages) as compared to positive control samples from the same staining series were registered. The CISH slides were analysed under an ordinary transmitted light microscope with × 40 and × 60 objectives (MN). In analysing CISH, the hybridisation results were registered in two classes (0 and +). The Her-2/neu proto-oncogene was considered to be amplified (+) if the copy number of the gene was six or more per cell (Tanner *et al*, 2001).

### Statistical analysis

The statistical analysis surveyed the routine clinico-pathological prognostic factors of the patient material. The data were collected according to the individually matched case–control design. This design ascertained that there was a controlled number of aggressive and control cancers that were matched with primary confounding factors. It followed from the matching that in the comparisons of cases and controls the confounders (used in the matching) were taken into account and there was no need to include the confounders into the statistical models. This led to more efficient estimation of associations of the prognostic variables of main interest. Moreover, it was not necessary to make assumptions about the association structure of confounders. Without matching it would have been possible to adjust the confounders equally efficiently only if the data had been enormously large. Even though the descriptive statistics in the results are shown separately for groups of aggressive and control cancers, the statistical analysis was based on stratification according to matched sets of patients. The prognostic associations of the variables with outcome of cancer were statistically analysed by conditional logistic regression analysis that takes into account the stratification due to the individual matching of the case and controls. In addition to *P*-values, the results were quantified using odds ratios (ORs) with 95% confidence intervals (CIs). Because the sample size was not large, *P*-values and CIs were calculated applying exact methods instead of the commonly used approximate asymptotic calculations (Breslow and Day, 1980; Hosmer and Lemeshow, 2000). Statistical computations were performed using SAS System for Windows, release 8.2/2001 and LogXact-4 for Windows, 2000 (Cytel Software Corporation)

## RESULTS

Summaries of IHC, CISH and histological grade are presented in Table 2

**Table 2:** Means (s.d.) of immunohistochemical stainings (percentage of positive cancer cells) and CISH (per cent of cases classified as positive) in the aggressive and control cancers

**Variable**	**Aggressive cancers**	**Control cancers**
Ki-67	26.7 (15.4)	16.5 (11.3)
p53	35.6 (38.3)	17.0 (26.8)
ER	49.0 (41.4)	70.6 (35.5)
PR	49.6 (38.8)	60.2 (35.4)
MAI	10.0 (9.2)	7.9 (9.5)
CISH (%)	6.1	4.0

Ki-67, p53, ER, PR=immunohistochemically detected positivity of Ki-67, p53, estrogen and progesterone hormone receptors, CISH=amplification of Her-2/neu.

. In univariate analysis (Table 3

**Table 3:** Results of unadjusted and adjusted exact conditional logistic regression analysis of all prognostic features presented by ORs with 95% CIs in the material of 66 T1N0M0 breast cancers

	**Unadjusted analysis**			**Adjusted for Ki-67**		
**Variable**	***P***	**OR**	**95% CI**	***P***	**OR**	**95% CI**
Ki-67	0.001	2.6	1.30–7.15			
p53	0.003	1.4	1.09–1.97	0.137	1.22	0.93–1.73
ER	0.020	0.8	0.71–0.98	0.219	0.9	0.75–1.07
CISH	0.069	3.9	0.78–24.70	0.683	1.4	0.20–9.17
PR	0.452	0.9	0.81–1.10			
*N*	0.432	1.1	0.82–1.61			
Histology	0.398	0.3	0.01–2.83			
Grade	0.177	2.0	0.74–6.74			

Ki-67, p53, ER, PR=immunohistochemically detected positivity of Ki-67, p53, estrogen and progesterone hormone receptors, CISH=amplification of Her-2/neu, *N*=number of examined axillary lymph nodes, histology=histological type (ductal or lobular), grade=WHO grade I–III.

), the strongest single feature predicting breast cancer death or recurrence was Ki-67 (*P*=0.002). Statistically significant prognostic features were also p53 (*P*=0.004) and ER (*P*=0.020). Her-2/neu was associated with a close-to-significant prognostic value (*P*=0.069). Instead, PR, ductal or lobular histological type, histological grade or number of investigated axillary lymph nodes were not statistically significant prognostic features in our material.

In statistical analysis, correlations could be expected between the strongest prognosticator, Ki-67, and other prognostic factors. In order to find out if ER, p53 or Her-2/neu had independent prognostic associations with aggressiveness of the disease, we analysed them by taking into account Ki-67. In a Ki-67-adjusted analysis, the directions of associations remained the same. However, ER, p53 or Her-2/neu were not found to give a significant addition to the prediction after taking into account Ki-67 (Table 3).

**Figure 1:**
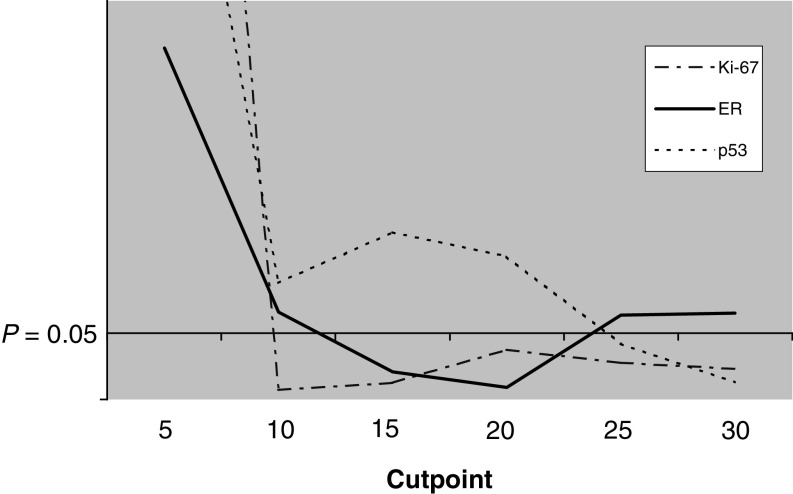
Prognostic value of immunopositivity of six cutpoints (5, 10, 15, 20, 25 and 30%) for Ki-67, ER and p53 in T1N0M0 breast cancer. The range of statistical significance (conditional logistic regression analysis) for each of the three features is shown under the line representing *P*-values equal or lower than 0.05. The most significant cutpoints are the lowest points of the curve at 10% for Ki-67, at 20% for ER and at 30% for p53.

In order to improve the routine clinical applicability of the prognostic features in T1N0M0 breast cancer, we set out to determine guidelines for interpreting IHC of Ki-67, p53 and ER. The guidelines were based on the present material testing the prognostic significance of six cutpoints of IHC (5, 10, 15, 20, 25 and 30%). In our material, the most significant cutpoint (Figure 1

) for Ki-67 was 10%. Patients with Ki-67 positivity in more than 10% of cancer cells were associated with an 11-fold odds of breast cancer death or recurrency compared to the patients with Ki-67 positivity in less than 10% of cancer cells (Table 4

**Table 4:** The most significant cutpoints for Ki-67, ER and p53 immunohistochemistry in our material of T1N0M0 breast cancer, and the relative risks (RRs) of breast cancer death or recurrence with *P*-values associated with each cutpoint

**Variable**	**Cutpoint (%)**	***P***	**RR**
Ki-67	10	0.007	11
ER	20	0.009	4
p53	30	0.013	10

). Correspondingly, patients with higher than 30% positivity in p53 were associated with a 10-fold odds for unfavourable outcome of disease. As to ER, the most significant cutpoint was at 20% of ER-positive cancer cells. However, in our material, patients with less than 20% of ER positivity were not significantly (*P*=0.092) associated with unfavourable outcome as compared with patients with more ER positivity (OR=3.63, 95% CI=0.79–22.45).

## DISCUSSION

In recent years, mammographic screening has made it possible to detect increasingly small invasive breast carcinomas and reduce the mortality of the disease (Anderson *et al*, 2000; Heimann *et al*, 2002; Louwman *et al*, 2002; Klemi *et al*, 2003; Tabar *et al*, 2003). Identifying aggressive disease in T1N0M0 breast cancer would give valuable information for planning adjuvant treatments.

The limited number of aggressive T1N0M0 breast cancer cases – as fortunate as it is for breast cancer patients – hampers clinical examination of the disease. In the two Finnish central hospitals involved in this study, a total of 21 T1N0M0 cases were reported with recurrent disease or breast cancer death during 1987–1997. In the present study, this problem was dealt with statistical techniques that are developed just in these kinds of situations. Firstly, with the individual matching, it was possible to control primary confounders and to ascertain that data from cases and controls corresponded to each level of the confounders. Secondly, with the conditional logistic regression analysis, it was possible to take into account the matching and perform the multivariate analysis. Thirdly, the matching (or stratification) made it possible to perform exact statistical calculations in the logistic analysis.

In addition to conventional morphological examination, ER, PR, Ki-67, p53 and Her-2/neu are highly established prognostic markers of invasive breast cancer. Also our analysis revealed that high Ki-67 and p53 immunopositivity, low ER immunopositivity and Her-2/neu oncogene amplification are the most reliable indicators of unfavourable outcome in T1N0M0 breast cancer. In multivariate analysis, high Ki-67 immunopositivity was the strongest individual prognosticator of breast cancer death or recurrence.

We further aimed at trying to intensify the value and applicability of Ki-67, p53 and ER immunohistochemistry by producing practical guidelines for their interpretation in the clinical setting. Based on the present material, we determined a range of clinical significance for each of the immunohistochemical prognostic features (Figure 1). In our material ,the range of optimal cutpoints for Ki-67 immunopositivity was 10% or above. Correspondingly, the cutpoint range for p53 was 30% or above and for ER 20%.

In literature, the criteria for determining Ki-67, p 53 and ER immunopositivity in breast cancer vary between 5 and 80% of cancer cells (Seymour *et al*, 1990; Jackson *et al*, 1990; Barnes and Millis, 1995; Goulding *et al*, 1995; Wishart *et al*, 2002). Also, a number of interpretation guidelines have been reported including categorical methods (Barnes and Millis, 1995), semiquantitative scoring systems (McCarty *et al*, 1985) and multivariate methods combining several interpretation features (Elston *et al*, 1998). Despite the convincing evidence on the prognostic value of IHC of Ki-67, p53 and ER in breast cancer, no universally accepted guidelines have been available for their interpretation this far. According to literature, reported poor specificity of ER immunohistochemistry in breast cancer can result in lower response rates to hormone therapy (McClelland *et al*, 1986; Robertson *et al*, 1992; Goulding *et al*, 1995). These facts emphasise the need for established clinically tested and uniformly practised interpretation guidelines for IHC in breast cancer. By using *in situ* hybridisation, we were able to avoid many of the difficulties encountered in the immunohistochemical analysis of Her2/neu (Tanner *et al*, 2001; Anttinen *et al*, 2003).

The small number of cases available for examination inevitably has its drawbacks on the conclusions of this paper. However, the patient material in the present study can be considered unique because of the accurate and careful selection of the control material as close as possible to perfect match between individual patient cases. The present results are consolidated by the appropriate study design of individually matched case–control approach. The uniformity and cohesiveness of the patient material emphasise the value of the obtained prognostic correlations.

Our paper emphasises the need for uniform prognostic methods in T1N0M0 breast cancer. In clinical pathology, no universally accepted cut points are available for the interpretation of immunohistochemical prognostic factors in breast cancer. Ki-67, p53 and ER immunohistochemistry, and Her-2/neu amplification analysis by CISH involves simple, standardised and established methods that can provide valuable prognostic information on T1N0M0 breast cancer. In the present paper, we summarise guidelines for identifying unfavourable outcome in T1N0M0 breast cancer. It is realistic to state that the cutpoints based on such limited data must be interpreted with care. However, in practice, a variety of cutpoints are clinically applied although none of them are based on careful analysis of any data – especially not of data for T1N0M0 tumours. The numerical cutpoints shown in the present study can give preliminary guidelines for clinical practice although our findings naturally need further verification. However, our results show that such guidelines can benefit individual breast cancer patients with more efficient and accurate treatment decisions.
